# Dual contribution of the gut microbiome to immunotherapy efficacy and toxicity: supportive care implications and recommendations

**DOI:** 10.1007/s00520-022-06948-0

**Published:** 2022-03-10

**Authors:** Hannah R. Wardill, Raymond J. Chan, Alexandre Chan, Dorothy Keefe, Samuel P. Costello, Nicolas H. Hart

**Affiliations:** 1 School of Biomedicine, The University of Adelaide, Adelaide, South Australia Australia; 2 Precision Medicine Theme, South Australian Health and Medical Research Institute, Adelaide, South Australia Australia; 3 Caring Futures Institute, College of Nursing and Health Sciences, Flinders University, Adelaide, South Australia Australia; 4 Division of Cancer Services, Princess Alexandra Hospital, Woolloongabba, Queensland Australia; 5 School of Pharmacy & Pharmaceutical Sciences, University of California Irvine, Irvine, CA USA; 6 Cancer Australia, Surry Hills, New South Wales Australia; 7 Adelaide Medical School, the University of Adelaide, Adelaide, South Australia Australia; 8 Department of Gastroenterology, Queen Elizabeth Hospital, Woodville South, South Australia Australia; 9 School of Medical and Health Sciences, Edith Cowan University, Joondalup, Western Australia Australia; 10 Institute for Health Research, University of Notre Dame Australia, Fremantle, Western Australia Australia

**Keywords:** Immune checkpoint inhibitors, Immunotherapy, Efficacy, Toxicity, Adverse events, Gut microbiome, Supportive cancer care

## Abstract

The efficacy of immune checkpoint inhibitors (immunotherapy) is increasingly recognized to be linked to the composition the gut microbiome. Given the high rates of resistance, interventions targeting the gut microbiome are now being investigated for its ability to improve the efficacy of immunotherapy. In light of recently published data demonstrating a strong correlation between the efficacy and toxicity of immunotherapy, there is a risk that efforts to enhance immunotherapy efficacy may be undermined by increases in immune-related adverse events (IrAEs) This is particularly important for microbial interventions aimed at increasing immunotherapy efficacy, with many microbes implicated in tumour response also linked to IrAEs, especially colitis. IrAEs have a profound impact on patient quality of life, causing physical, psychosocial, and financial distress. Here, we outline strategies at the discovery, translational, and clinical research phases to ensure the impact of augmenting immunotherapy efficacy is approached in a manner that considers adverse implications. Adopting these strategies will ensure that our ongoing efforts to overcome immunotherapy resistance are not impacted by unacceptable toxicity.

## Introduction

The discovery of the inhibitory immune checkpoint molecules programmed cell death protein 1 and its ligand (PD-1 and PD-L1), as well as the cytotoxic T lymphocyte–associated antigen 4 (CTLA-4), has undeniably advanced the landscape of cancer control [1]. Monoclonal antibodies that target PD-1, PD-L1, and CTLA-4 confer significant and often durable clinical responses and have set new standards of care across a variety of malignant diseases (especially melanoma and lung cancers) [2–4]. Despite these advances, resistance to immunotherapy remains a significant challenge and an area of intense investigation to devise strategies that facilitate or potentiate immunotherapeutic response [5]. In particular, the contribution of the gut microbiome (the collection of micro-organisms that reside in the gut) has gained significant momentum, with distinct microbial signatures predicting patient responses. While certainly an exciting advance in overcoming immunotherapy resistance, comparable microbial traits appear to also regulate treatment toxicity. As such, there is a risk that efforts to enhance immunotherapy efficacy may be undermined by increases in immune-related adverse events (IrAEs). Here, we discuss these potential consequences and outline supportive care strategies to minimize adverse effects while enhancing immunotherapy efficacy.

## Gut microbiome and immunotherapy efficacy

The gut microbiome, the ecosystem of micro-organisms (bacteria, viruses and fungi) and their metabolic products in the gut, has a profound influence on the host’s immune system, governing the delicate balance between tolerance and initiation of appropriate response to antigens [6]. The unique composition of an individual’s gut microbiome is now understood to effect immune tone and risk of immune-driven disease [7]. It is therefore unsurprising that the gut microbiome is linked with immunotherapy treatment response and, by extension, resistance [8]. Central to this observation is the detrimental impact of antibiotics on immunotherapy efficacy. A recent meta-analysis reported progression-free and overall survival were negatively impacted by antibiotic use [9], suggesting that disruption of the gut microbiome’s natural, eubiotic state by antibiotics dampens anti-cancer immunity, subsequently leading to poorer clinical outcomes [10].

The concept that a disrupted gut microbiome (namely by antibiotics) impairs responsiveness to immunotherapy suggests that a rich and diverse microbiome is important for immunotherapy efficacy [11]. In fact, promising data from two small clinical studies (1 study *n* = 15 with clinical benefit in 6 of 15 patients, and the other study *n* = 10) [12, 13] show faecal microbiota transplant (FMT)—a method that enables the composition of the gut microbiome to be changed by transferring the entire gut microbiota from one host (donor) to another (recipient)—can improve immunotherapy efficacy. FMT from individual, long-term responders (R) has been shown, in two separate studies, to overcome resistance and promote response in metastatic [12] and refractory melanoma [13]. In parallel, optimizing immunotherapy efficacy through more targeted microbial manipulation has been an area of enthusiastic investigation in preclinical models [10]. For example, the efficacy of adoptive cell therapy was shown to be enhanced by selectively targeting and eliminating specific microbes belonging to the *Bacteroidetes* phylum [14]. Similarly, administration of the commensal microbe, *Bifidobacterium* spp., has been shown to enhance the efficacy of a PD-L1 therapy in a rodent model of melanoma [15]. Mechanistically, this was hypothesized to be driven by the microbiome’s capacity to dictate peripheral Th1-skewed inflammatory responses (e.g. increased CD8 + dendritic cells and peripheral IL-12 concentrations) resulting in increased accumulation of T-cells within the tumour, thus enhancing anti-tumour efficacy [16]. While these data point to an exciting opportunity to enhance anti-tumour responses via microbial intervention (e.g. FMT or specific microbial delivery), they also raise important questions regarding the adverse effects of promoting peripheral inflammation. Given aberrant inflammation drives numerous adverse effects of anti-cancer drugs, including immunotherapy, there is a possibility that enhancing efficacy will also increase the risk of toxicity [17, 18].

## Gut microbiome and immunotherapy toxicity

Immunotherapy is associated with a range of adverse toxicities (e.g. colitis, hepatitis, pneumonia, fatigue) that results from over-activation of the immune system (termed immune-related adverse events, IrAEs) [19–22]. In line with the evidence for immunotherapy efficacy, the gut microbiome also appears to modulate the incidence and severity of IrAEs [23]. Of particular importance, evidence exploring the contribution of the microbiome to immunotherapy response highlights significant overlap in the microbial phenotypes that govern both efficacy and toxicity. For example, while a microbiome enriched for the *Faecalibacterium* genus and other *Firmicute* phyla conferred a more favourable anti-tumour response in patients with melanoma (increased progression free and overall survival), these patients were also at an increased risk of colitis [24]. In a different study, a microbiome enriched for the *Gemmiger formicilis* genus was associated with both the efficacy and toxicity of immunotherapy used to treat melanoma [24, 25]. Numerous clinical trials of immune checkpoint inhibitors have also highlighted this clinical challenge, reporting positive associations between efficacy and the incidence/severity of IrAEs (clinical trials summarized in [26]). This was clearly demonstrated in a recent meta-analysis which included data from 30 studies (*n* = 4971)—the majority of which did not include microbiome data—where patients with IrAEs had increased overall (OS) and progression-free survival (PFS) compared to those who did not develop IrAEs (OS: hazard ratio (HR), 0.54, 95% confidence interval (CI), 0.45–0.65; *p* < 0.001; PFS: HR, 0.52, 95% CI, 0.44–0.61, *p* < 0.001) [26].

While further work is needed to dissect the complex interplay between efficacy and toxicity, the potential contribution of the microbiome to the efficacy and toxicity of immunotherapy highlights the dynamic interplay that exists between these opposing treatment outcomes, which are ultimately governed by the same immune-dependent mechanisms against different cell populations (i.e. healthy vs. tumour). Conceptually, the idea that efficacy and toxicity are intimately linked is not new nor surprising. For instance, when using targeted kinase inhibitors (TKIs), acute toxicities are indicative of active anti-tumour responses (e.g. TKI rash) [27–30]. Similarly, traditional anti-cancer agents (e.g. chemotherapy and radiotherapy) are well understood to kill tumour cells and healthy tissue by indiscriminate and irreversible DNA damage, resulting in cell death. This overlap has, in some cases, presented as a major obstacle, with new supportive care interventions aiming to minimize off-target cytotoxicity also impairing the efficacy of chemotherapy [31]. We now suggest that the impact of enhancing immunotherapy efficacy on IrAE risk needs to be appropriately recognized to ensure immunotherapy can be optimized, but not at the cost of the patient’s health and wellbeing.

## Our recommendations

**Fig. 1:**
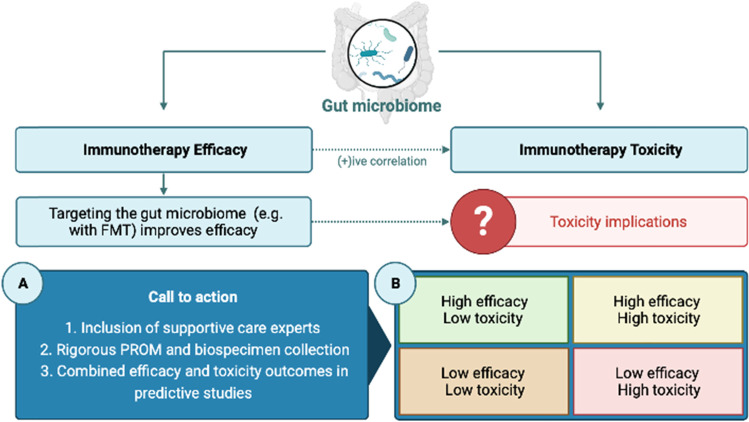
The dual contribution of the gut microbiome to the efficacy and toxicity of immunotherapy. Microbial signatures that predict efficacy often parallel those that predict IrAEs, mirroring clinical data that show a positive association between efficacy and toxicity. Attempts to enhance efficacy through microbial intervention must therefore consider the implications for heightened toxicity. Our call to action (**A**) reinforces the critical need to include supportive care experts from early in the research pipeline and rigorous PROM/biospecimen collection to ensure new attempts to enhance immunotherapy efficacy do not negatively impact patient health and wellbeing. We also highlight (**B**) that attempts to identify microbial predictors of response need to be performed with more granular stratification of patient outcomes that includes efficacy and toxicity outcomes. This would identify the “optimal responder”, that is, a responder with mild and manageable IrAEs. Image generated by BioRender

In our continued attempts to increase the efficacy of immunotherapy by targeting the gut microbiome, the potential of exacerbating toxicity must be acknowledged and carefully considered to ensure patient quality of life remains intact. This requires symptom management and supportive cancer care experts be involved in the early stages of translational research to guide the appropriate evaluation of toxicity in preclinical studies and ensure clinical studies measure adverse events with the appropriate tools that capture the patient experience [32]. The inclusion of patient-reported outcome measures (PROMs) is particularly critical as evidence clearly demonstrates that clinician-reported AEs grossly underestimate the impact of symptoms on patient quality of life [33]. With a plethora of electronic-PROM assessment tools that overcome logistical challenges of PROM collection, inclusion of appropriate and consistent PROMs should be routinely encouraged and mandated. Such incorporation of PROMs in all translational research and clinical care settings is critical for building a comprehensive evidence base for the intricate relationships highlighted in this article, ultimately informing treatment decision-making and supportive care interventions (Fig. 1A).

Additionally, we recommend a more granular approach to predicting immunotherapy outcomes. A limitation of currently available, published datasets is that predictive microbial signatures have been linked with dichotomous outcomes (e.g. responders vs. non-responders, colitis vs. no colitis). However, within dichotomous outcomes, there is tremendous variability that warrants further investigation. For example, there are subsets of patients that will be one of responders with severe toxicity; responders with none-to-mild toxicity; non-responders with severe toxicity; and non-responders with none-to-mild toxicity. Identifying the microbial signatures of these cohorts will be insightful, and a more valid approach to identify the “optimal responder” (i.e. responders with high efficacy and low toxicity; Fig. 1B). It is likely that limitations in microbiome-based modelling may have required dichotomous outcomes to be analyzed to date. However, there is an increasing focus on the use of artificial-intelligence and machine learning approaches that are capable of handling more complex datasets [34]. In addition, deploying metagenomic sequencing paired with bacterial culturing in observational or interventional microbiome studies allows more precise analysis of microbial populations at strain level resolution. This approach along with metabolomics, shotgun metagenomics, and metatranscriptomics can help elucidate the host-microbe mechanisms that drive efficacy and toxicity, individually and collectively [35]. By leveraging these emerging technologies and ensuring appropriate biospecimens (e.g. pre-treatment stool samples) with paired IrAEs are routinely and consistently collected from patients (e.g. clinical trial participants), we can identify optimal responders and dissect the host-microbe mechanisms that dictate efficacy in a manner that does not impact toxicity, ultimately teasing apart this very delicate relationship.

## Data Availability

Not applicable.
